# Supplementary material for: Meiotic, genomic and evolutionary properties of crossover distribution in Drosophila yakuba
Source: PLoS Genet. 2022 Mar 23;18(3):e1010087. doi: 10.1371/journal.pgen.1010087 (PMC8979470; doi:10.1371/journal.pgen.1010087)
Supplement: S1 Table — (PDF) [file pgen.1010087.s001.pdf]

S1 Table. Observed number of meiotic events and crossovers for each of the three crosses of *D. yakuba* analyzed in this study.

	TZ043 x *TZ020*				
	X	*2L*	*2R*	*3L*	*3R*
NCO[1]	117	244	207	199	202
1CO	290	232	147	237	273
2CO	68	46	31	79	38
3CO	27	1	11	7	5
4CO	0	0	1	0	1
Average CO / chromatid	1.01	0.63	0.62	0.80	0.71

	Sn20 x *Sn17*				
	X	*2L*	*2R*	*3L*	*3R*
NCO[1]	168	264		211	202
1CO	281	220		278	278
2CO	47	34		72	39
3CO	18	0		3	3
4CO	0	0		0	0
Average CO / chromatid	0.83	0.56		0.76	0.70

	Rain5 x *Cost1235.2*				
	X	*2L*	*2R*	*3L*	*3R*
NCO[1]	162	327	256	284	279
1CO	330	256	165	266	278
2CO	85	36	19	58	30
3CO	29	1	3	7	7
4CO	0	0	1	0	0
Average CO / chromatid	0.97	0.53	0.49	0.66	0.60

[1] NCO: no crossovers, 1CO: single crossover, 2CO: double crossover, 3CO: triple crossover, 4CO: 4 crossovers in a single chromatid.
