# Supplementary material for: Meiotic, genomic and evolutionary properties of crossover distribution in Drosophila yakuba
Source: PLoS Genet. 2022 Mar 23;18(3):e1010087. doi: 10.1371/journal.pgen.1010087 (PMC8979470; doi:10.1371/journal.pgen.1010087)
Supplement: S2 Table — (PDF) [file pgen.1010087.s002.pdf]

S2 Table. Centromere and telomere effect in *D. yakuba* based on the study of the most centromere- and telomere-proximal 1/3 region of each chromosome arm.

	X	*2L*	*2R*	*3L*	*3R*
Centromere	0.127	$<1x10^{-7}$	$<1x10^{-7}$	$<1x10^{-7}$	$<1x10^{-7}$
Telomere	$<1x10^{-7}$	1	1	1	0.999

[1] Values indicate the proportion of trials with less crossovers than those observed based on random distribution of crossovers along the entire chromosome arm (see Materials and Methods).
