# Supplementary material for: Meiotic, genomic and evolutionary properties of crossover distribution in Drosophila yakuba
Source: PLoS Genet. 2022 Mar 23;18(3):e1010087. doi: 10.1371/journal.pgen.1010087 (PMC8979470; doi:10.1371/journal.pgen.1010087)
Supplement: S3 Table — (PDF) [file pgen.1010087.s003.pdf]

S3 Table. Proportion of chromosome arms showing centromere and telomere effect in *D. yakuba* and *D. melanogaster.*

		Centromere Effect				
		Proportion of Chromosome[1]				
	Significance level	*X*	*2L*	*2R*	*3L*	*3R*
D. yakuba	$P < 1{\times}10^{-6}$	0.12	0.43	0.12	0.35	0.47
	$P < 0.01$	0.13	0.50	0.15	0.39	0.52
D. melanogaster r6	$P < 1{\times}10^{-6}$	0.12	0.08	0.22	0.24	0.25
	$P < 0.01$	0.13	0.28	0.26	0.29	0.27
D. melanogaster r5.3	$P < 1{\times}10^{-6}$	0.09	0.27	0.18	0.21	0.16
	$P < 0.01$	0.13	0.29	0.27	0.24	0.27

		Telomere Effect				
		Proportion of Chromosome				
	Significance level	*X*	*2L*	*2R*	*3L*	*3R*
D. yakuba	$P < 1{\times}10^{-6}$	0.12	0.04	0.03	0.00	0.04
	$P < 0.01$	0.15	0.06	0.06	0.04	0.04
D. melanogaster r6	$P < 1{\times}10^{-6}$	0.11	0.04	0.00	0.00	0.01
	$P < 0.01$	0.12	0.04	0.00	0.03	0.02
D. melanogaster r5.3	$P < 1{\times}10^{-6}$	0.12	0.00	0.02	0.00	0.01
	$P < 0.01$	0.13	0.05	0.02	0.04	0.03

[1] Proportion of the chromosome with significant reduction in crossovers based on a sliding window study of a 1-Mb region and a step of 100 kb towards the center of the chromosome arm (see Materials and Methods). For *D. melanogaster,* genome annotations r5.3 and r6 were analyzed. Two levels of significance are shown.
