# Supplementary material for: Meiotic, genomic and evolutionary properties of crossover distribution in Drosophila yakuba
Source: PLoS Genet. 2022 Mar 23;18(3):e1010087. doi: 10.1371/journal.pgen.1010087 (PMC8979470; doi:10.1371/journal.pgen.1010087)
Supplement: S4 Table — (PDF) [file pgen.1010087.s004.pdf]

S4 Table. Satellite repeats in euchromatic and heterochromatic regions of *D. yakuba* and *D. melanogaster*[1].

	D. yakuba		*D. melanogaster*			
Satellite	Euchrom.	Heterochrom.	Euchrom.	Heterochrom.	*P*	Direction
AATAT	68091	8090	393911	92612	$<1.8\times10^{-322}$	M
AAGAG	19	0	723191	108712	$<1.8\times10^{-322}$	M
AACATAGAAT	0	0	44637	81886	$<1.8\times10^{-322}$	M
AAGAT	0	0	95522	88427	$<1.8\times10^{-322}$	M
ACCAGTACGGG	0	0	395	734	1.64×10^{-247}	M
AATAG	0	0	52896	39507	$<1.8\times10^{-322}$	M
C	451494	20434	297575	25090	$<1.8\times10^{-322}$	Y
AAACAAT	51037	5625	6849	58902	8.83×10^{-149}	M
AAAATAT	14189	1919	6868	6794	1.28×10^{-45}	Y
AG	5055	217	18402	7976	$<1.8\times10^{-322}$	M
AAT	103831	7963	75269	2380	$<1.8\times10^{-322}$	Y
AT	15149	509	56724	6425	$<1.8\times10^{-322}$	M
AGAT	4717	61	81494	10024	$<1.8\times10^{-322}$	M
AC	7824	29	5829	305	7.28×10^{-48}	Y
AATATAT	2983	0	75396	6956	$<1.8\times10^{-322}$	M
AAATTAT	1791	55	7068	1254	$<1.8\times10^{-322}$	M
AATAATAT	2076	0	25147	2323	$<1.8\times10^{-322}$	M
CG	190	28	0	35	1.24×10^{-30}	Y
AGAGGG	154	0	39	59	4.19×10^{-04}	Y
AAAACAATAAACAAT	110	8	9	60	3.39×10^{-04}	Y
AAAACAAT	119	14	9	60	6.70×10^{-06}	Y
AAATAT	1541	47	1766	256	5.07×10^{-13}	M
AAG	695	55	4715	1209	$<1.8\times10^{-322}$	M
AACAAT	124	39	0	29	4.02×10^{-22}	Y
AAATATAT	704	0	4098	342	1.22×10^{-187}	M
AAATAAT	239	0	6620	1384	$<1.8\times10^{-322}$	M
AAAAG	155	0	6649	1100	$<1.8\times10^{-322}$	M
AAC	599	127	642	53	1.90×10^{-17}	Y
AAAAATAT	123	0	14	7	7.99×10^{-25}	Y
AAAAAT	1034	12	60	10	$<1.8\times10^{-322}$	Y
AATACC	37398	64157	9	0	$<1.8\times10^{-322}$	Y
AATATATAT	101	0	202	19	2.27×10^{-11}	M
AATAGAATAT	37279	4786	0	0	$<1.8\times10^{-322}$	Y
AAAGAAAT	14740	7212	0	0	$<1.8\times10^{-322}$	Y
AATACAT	13673	6907	0	0	$<1.8\times10^{-322}$	Y
ACTCTAT	2087	0	0	0	$<1.8\times10^{-322}$	Y

S4 Table. – continued

Satellite	*D. yakuba* Euchrom.	*D. yakuba* Heterochrom.	*D. melanogaster* Euchrom.	*D. melanogaster* Heterochrom.	*P*	Direction
ACTGCT	1961	0	0	0	$<1.8\times10^{-322}$	Y
ACATAT	1403	0	0	0	4.68×10^{-307}	Y
AATAGC	1274	0	0	0	5.05×10^{-279}	Y
ACCCAT	1226	0	0	0	1.36×10^{-268}	Y
AAAATAAAT	1011	14	0	0	6.61×10^{-225}	Y
AAGTAC	773	0	0	0	4.00×10^{-170}	Y
ACAGACGG	680	0	0	0	6.68×10^{-150}	Y
AAATAAATT	617	34	0	0	1.35×10^{-143}	Y
AAAAT	245	304	0	0	2.08×10^{-121}	Y
AACTAC	547	0	0	0	5.66×10^{-121}	Y
AAAAATT	446	17	0	0	1.07×10^{-102}	Y
AAATATG	463	0	0	0	1.07×10^{-102}	Y
AACAT	385	0	0	0	1.01×10^{-85}	Y
AACCT	252	0	0	0	9.52×10^{-57}	Y
AAACAG	227	0	0	0	2.69×10^{-51}	Y
ATCC	229	0	0	0	9.85×10^{-52}	Y
AAACATAC	215	0	0	0	1.11×10^{-48}	Y
AAGTAG	196	0	0	0	1.56×10^{-44}	Y
AAAAAAT	54	141	0	0	2.58×10^{-44}	Y
ACATCC	192	0	0	0	1.16×10^{-43}	Y
ACCACT	182	0	0	0	1.77×10^{-41}	Y
ACCTCC	173	0	0	0	1.64×10^{-39}	Y
ATATC	158	0	0	0	3.09×10^{-36}	Y

[1] Number of repeats for euchromatic and heterochromatic regions in *D. yakuba* and *D. melanogaster* based on the study of PacBio reads that do and do not map to the corresponding reference genomes, respectively. Centromeric and centromere-proximal satellite repeats in *D. melanogaster* are underlined [1]. *P*, χ^2 test for the difference in the ratios of euchromatic and heterochromatic satellite presence between species. Direction indicates which species has more satellites: M, *D. melanogaster*; Y, *D. yakuba*.

References

1. Chang C-H, Chavan A, Palladino J, Wei X, Martins NMC, Santinello B, et al. Islands of retroelements are major components of Drosophila centromeres. PLoS biology. 2019;17(5):e3000241. doi: 10.1371/journal.pbio.3000241.
