# Supplementary material for: Meiotic, genomic and evolutionary properties of crossover distribution in Drosophila yakuba
Source: PLoS Genet. 2022 Mar 23;18(3):e1010087. doi: 10.1371/journal.pgen.1010087 (PMC8979470; doi:10.1371/journal.pgen.1010087)
Supplement: S5 Table — (PDF) [file pgen.1010087.s005.pdf]

S5 Table. Number of satellite repeats per Mb in heterochromatic and euchromatic PacBio reads of *D. yakuba* and *D. melanogaster*.

	D. yakuba	*D. melanogaster*	Ratio *D. yak./D. mel.*
Heterochromatin	601.4	14867.9	24.7
Euchromatin	185.2	756.6	4.1
Ratio Het./Eu.	3.2	19.7	
