# Supplementary material for: Meiotic, genomic and evolutionary properties of crossover distribution in Drosophila yakuba
Source: PLoS Genet. 2022 Mar 23;18(3):e1010087. doi: 10.1371/journal.pgen.1010087 (PMC8979470; doi:10.1371/journal.pgen.1010087)
Supplement: S6 Table — (PDF) [file pgen.1010087.s006.pdf]

S6 Table. Tetrad analysis and estimates of *E* values[1] for *D. yakuba* and *D. melanogaster*.

D. yakuba (this study)					
	E_0	E_1	E_2	E_3	E_4
All	-0.005	0.687	0.190	0.126	0.002
Autosomes	0.059	0.621	0.269	0.048	0.003
X	-0.202	0.812	0.166	0.126	0.098

D. melanogaster (WGS) [2]					
	E_0	E_1	E_2	E_3	E_4
All	0.112	0.680	0.200	0.008	0.000
Autosomes	0.110	0.697	0.183	0.010	0.000
X	0.122	0.614	0.264	0.000	0.000

D. melanogaster (visible markers) [3]					
	E_0	E_1	E_2	E_3	E_4
All	0.161	0.746	0.092	0.001	0.000
Autosomes	0.186	0.724	0.090	0.000	0.000
X	0.146	0.759	0.094	0.001	0.000

[1] Estimates of *E* for *D. yakuba* and *D. melanogaster* following [1] with unrestricted values for E_r. E_0: tetrads that do not undergo crossing over, E_1: tetrads experiencing 1 CO, E_2: tetrads experiencing 2 COs, E_3: tetrads experiencing 3 COs, and E_4: tetrads experiencing 4 COs. Values for *D. yakuba* do not include data from chromosome arm *2R*. [2] *D. melanogaster* data from whole genome sequencing/genotyping (WGS) datasets from [2] not including *2R* in order to allow a direct comparison with *D. yakuba*. [3] *D. melanogaster* data, not including *2R,* from the study of visible markers from [3-5] (see S11 Table).

References

1. Weinstein A. The theory of multiple-strand crossing over. Genetics. 1936;21(3):155-99.
2. Miller DE, Smith CB, Kazemi NY, Cockrell AJ, Arvanitakas AV, Blumenstiel JP, et al. Whole-genome analysis of individual meiotic events in *Drosophila melanogaster* reveals that noncrossover gene

conversions are insensitive to interference and the centromere effect. Genetics. 2016;203(1):159-71. doi: 10.1534/genetics.115.186486.

3. Hatkevich T, Kohl KP, McMahan S, Hartmann MA, Williams AM, Sekelsky J. Bloom Syndrome Helicase Promotes Meiotic Crossover Patterning and Homolog Disjunction. Current Biology. 2017;27(1):96-102. doi: https://doi.org/10.1016/j.cub.2016.10.055
4. Baker BS, Carpenter ATC. Genetic analysis of sex chromosomal meiotic mutants in *Drosophila melanogaster*. Genetics. 1972;71(2):255-86.
5. Parry DM. A meiotic mutant affecting recombination in female *Drosophila melanogaster*. Genetics. 1973;73(3):465-86.
