# Supplementary material for: Meiotic, genomic and evolutionary properties of crossover distribution in Drosophila yakuba
Source: PLoS Genet. 2022 Mar 23;18(3):e1010087. doi: 10.1371/journal.pgen.1010087 (PMC8979470; doi:10.1371/journal.pgen.1010087)
Supplement: S7 Table — (PDF) [file pgen.1010087.s007.pdf]

S7 Table. Transposable Elements (TEs) showing a significant difference in copy number between *D. yakuba* and *D. melanogaster*.

TE name	*D. yakuba*	*D. melanogaster*	P_{adj} [1]	Direction[2]
INE-1	3154	1812	1.26×10^{-78}	Y
1360	95	269	1.27×10^{-17}	M
Osvaldo	91	28	1.29×10^{-6}	Y
Dsub\SGM	95	33	7.10×10^{-6}	Y
diver2	119	52	4.98×10^{-5}	Y
HMS-Beagle2	12	43	0.005	M
HMS-Beagle	10	38	0.009	M
Baggins	11	39	0.012	M
297	34	73	0.026	M
Max-element	99	53	0.031	Y
Hobo	64	29	0.045	Y

[1] P_{adj} is the result of a χ^2 test for a difference in abundance between the two species after applying the Benjamini-Hochberg correction. Only TEs with a significant difference between *D. yakuba* and *D. melanogaster* are shown. [2] Direction indicates which species has more copies. M, *D. melanogaster*; Y, *D. yakuba*.
