# Supplementary material for: Meiotic, genomic and evolutionary properties of crossover distribution in Drosophila yakuba
Source: PLoS Genet. 2022 Mar 23;18(3):e1010087. doi: 10.1371/journal.pgen.1010087 (PMC8979470; doi:10.1371/journal.pgen.1010087)
Supplement: S8 Table — (PDF) [file pgen.1010087.s008.pdf]

S8 Table. Spearman's ρ correlation between TE abundance and crossover rate (cM/Mb) in *D. yakuba* and *D. melanogaster*[1].

	D. yakuba			*D. melanogaster*			Fisher's z[2]
	n	ρ	*P* value	n	ρ	*P* value	*P* value
all TEs	6210	-0.5431	5.2×10^{-41}	4765	-0.3543	1.6×10^{-15}	$<8\times10^{-15}$
only INE-1	3154	-0.5847	9.8×10^{-49}	1812	-0.3777	1.4×10^{-17}	$<8\times10^{-15}$

[1] Study based on non-overlapping 200 kb regions. [2] Fisher's *z*-transformed analysis to test the similarity of ρ in *D. yakuba* and *D. melanogaster*.
