# Supplementary material for: Meiotic, genomic and evolutionary properties of crossover distribution in Drosophila yakuba
Source: PLoS Genet. 2022 Mar 23;18(3):e1010087. doi: 10.1371/journal.pgen.1010087 (PMC8979470; doi:10.1371/journal.pgen.1010087)
Supplement: S9 Table — (PDF) [file pgen.1010087.s009.pdf]

S9 Table. Spearman's ρ correlation between abundance of TE classes and crossover rate (cM/Mb) in *D. yakuba* and *D. melanogaster*[1].

	All Chromosomes						
	D. yakuba			*D. melanogaster*			Fisher's *z*
TE Class	N	ρ	*P*	N	ρ	*P*	*P*
LTR	1677	-0.285	4.4×10^{-11}	1781	-0.277	$7.67E^{-10}$	n.s.
TIR	309	-0.190	1.4×10^{-05}	177	-0.329	$1.66E^{-13}$	n.s.
Line-like	587	-0.188	1.7×10^{-05}	538	-0.263	$5.59E^{-09}$	n.s.
SINE	3154	-0.585	9.8×10^{-49}	1812	-0.378	$1.37E^{-17}$	$<8x10^{-15}$
non-LTR	261	-0.298	5.0×10^{-12}	295	-0.276	$8.76E^{-10}$	n.s.
Foldback	43	-0.186	2.2×10^{-5}	69	-0.124	0.0070	n.s.
IR element	82	-0.311	4.4×10^{-13}	56	-0.285	$2.57E^{-10}$	n.s.
MITE	96	-0.303	2.0×10^{-12}	30	-0.323	$5.01E^{-13}$	n.s.
DNA	1217	-0.289	2.0×10^{-11}	178	-0.326	$3.13E^{-13}$	n.s.
RNA	4933	-0.577	2.9×10^{-47}	4581	-0.356	$1.20E^{-15}$	$<8x10^{-15}$

	Autosomes						
	D. yakuba			*D. melanogaster*			Fisher's *z*
TE Class	N	ρ	*P*	N	ρ	*P*	*P*
LTR	1301	-0.313	1.4×10^{-10}	1391	-0.325	1.4×10^{-10}	n.s.
TIR	256	-0.223	6.3×10^{-06}	157	-0.347	6.4×10^{-12}	n.s.
Line-like	458	-0.231	2.9×10^{-06}	489	-0.324	1.6×10^{-10}	n.s.
SINE	2813	-0.612	1.7×10^{-42}	1328	-0.468	2.3×10^{-21}	9.4×10^{-10}
non-LTR	227	-0.325	2.5×10^{-11}	204	-0.282	3.1×10^{-08}	n.s.
Foldback	38	-0.174	4.7×10^{-04}	40	-0.138	0.0078	n.s.
IR element	77	-0.309	2.5×10^{-10}	42	-0.354	2.1×10^{-12}	n.s.
MITE	84	-0.295	1.8×10^{-09}	15	-0.342	1.3×10^{-11}	n.s.
DNA	998	-0.330	1.3×10^{-11}	198	-0.382	2.5×10^{-14}	n.s.
RNA	4257	-0.613	1.2×10^{-42}	3469	-0.429	4.7×10^{-18}	$<8\times10^{-15}$

S9 Table. – continued

	Chromosome *X*						
	D. yakuba			*D. melanogaster*			Fisher's *z*
TE Class	N	ρ	*P*	n	ρ	*P*	*P*
LTR	376	-0.136	n.s.	390	-0.136	n.s.	n.s.
TIR	53	-0.125	n.s.	20	-0.325	0.0009	n.s.
Line-like	129	-0.231	0.0125	49	-0.085	n.s.	n.s.
SINE	341	-0.379	$2.7{\times}10^{-05}$	484	-0.297	0.0021	n.s.
non-LTR	34	-0.152	n.s.	91	-0.301	0.0018	n.s.
Foldback	12	-0.228	0.0147	15	-0.130	n.s.	n.s.
IR element	219	-0.246	0.0078	49	-0.225	0.0210	n.s.
MITE	736	-0.337	0.0002	1112	-0.260	0.0073	n.s.
DNA	376	-0.136	n.s.	390	-0.136	n.s.	n.s.
RNA	53	-0.125	n.s.	20	-0.325	0.0009	n.s.

[1] Classes with less than 10 alignments in at least one species are not shown.
