# Supplementary material for: Meiotic, genomic and evolutionary properties of crossover distribution in Drosophila yakuba
Source: PLoS Genet. 2022 Mar 23;18(3):e1010087. doi: 10.1371/journal.pgen.1010087 (PMC8979470; doi:10.1371/journal.pgen.1010087)
Supplement: S10 Table — (PDF) [file pgen.1010087.s010.pdf]

S10 Table. Enrichment analysis of individual motifs near crossover events in *D. yakuba*[1].

Motif (M[2])	*P*-value 5-kb	3-kb	1-kb
$[A]_N$	$<3.3\times10^{-308}$	1.7×10^{-71}	3.19×10^{-36}
A (M3)	1.5×10^{-123}	2.9×10^{-08}	3.19×10^{-03}
B (M5)	3.4×10^{-130}	1.07×10^{-32}	3.89×10^{-27}
C (M10)	1.07×10^{-92}	1.01×10^{-44}	5.16×10^{-70}
D (M12)	1.1×10^{-107}	2.22×10^{-09}	1.69×10^{-03}
$[CA]_N$	$<3.3\times10^{-308}$	1.42×10^{-65}	1.41×10^{-05}
E (M1)	$<3.3\times10^{-308}$	9.75×10^{-36}	n.s.
F (M4)	4.94×10^{-59}	6.07×10^{-32}	4.98×10^{-08}
$[TA]_N^{*}$ (M7)	5.17×10^{-92}	5.07×10^{-45}	1.57×10^{-04}
$[GCA]_N$ (M2)	$<3.3\times10^{-308}$	1×10^{-199}	4.41×10^{-03}
$[CYCYYY]_N$ (M6)	1.47×10^{-60}	2.26×10^{-40}	9.62×10^{-05}

[1] Probabilities were obtained by comparing the number of times a motif is identified by FIMO in sequences containing a crossover event and expectations based on sequences of equivalent length randomly chosen across the genome (see **S1 Text**). Three sequence datasets were analyzed based on the distance between diagnostic SNPs around a crossover event (5-kb or less, 3-kb or less, and 1-kb or less). Shaded rows represent the analysis of the combined motifs within the $[A]_N$ and $[CA]_N$ class. [2] M indicates the motif notation from [1]. See **S3 Fig** for details.

References

1. Adrian AB, Corchado JC, Comeron JM. Predictive Models of Recombination Rate Variation across the *Drosophila melanogaster* Genome. Genome Biology and Evolution. 2016;8(8):2597-612. doi: 10.1093/gbe/evw181.
