# Supplementary material for: Meiotic, genomic and evolutionary properties of crossover distribution in Drosophila yakuba
Source: PLoS Genet. 2022 Mar 23;18(3):e1010087. doi: 10.1371/journal.pgen.1010087 (PMC8979470; doi:10.1371/journal.pgen.1010087)
Supplement: S11 Table — (PDF) [file pgen.1010087.s011.pdf]

S11 Table. Data used to study tetrad frequencies in *D. melanogaster*.

	Chromosome								
CO Class	*X*[1]	*2L*[1]	*2R*[1]	*3L*[1]	*3R*[1]	*X*[2]	*2L*[3]	*2L*[2]	*2R*[4]
NCO	97	99	101	97	99	1,015	6,308	2,376	1,698
1CO	86	88	90	84	86	964	4,909	1,758	1,150
2CO	13	9	5	15	10	197	276	88	73
3CO	0	0	0	0	1	3	2	0	0
Total	196	196	196	196	196	2,179	11,495	4,222	2,921

[1] Whole genome sequencing/genotyping (WGS) from [1],[2] visible markers from [2], [3] visible markers from [3], [4] visible markers from [4]. NCO, noncrossover; 1CO, single crossover; 2CO, double crossover; 3CO, triple crossover.

References

1. Miller DE, Smith CB, Kazemi NY, Cockrell AJ, Arvanitakas AV, Blumenstiel JP, et al. Whole-genome analysis of individual meiotic events in *Drosophila melanogaster* reveals that noncrossover gene conversions are insensitive to interference and the centromere effect. Genetics. 2016;203(1):159-71. doi: 10.1534/genetics.115.186486.
2. Hatkevich T, Kohl KP, McMahan S, Hartmann MA, Williams AM, Sekelsky J. Bloom Syndrome Helicase Promotes Meiotic Crossover Patterning and Homolog Disjunction. Current Biology. 2017;27(1):96-102. doi: https://doi.org/10.1016/j.cub.2016.10.055
3. Baker BS, Carpenter ATC. Genetic analysis of sex chromosomal meiotic mutants in *Drosophila melanogaster*. Genetics. 1972;71(2):255-86.
4. Parry DM. A meiotic mutant affecting recombination in female *Drosophila melanogaster*. Genetics. 1973;73(3):465-86.
