# Supplementary material for: Meiotic, genomic and evolutionary properties of crossover distribution in Drosophila yakuba
Source: PLoS Genet. 2022 Mar 23;18(3):e1010087. doi: 10.1371/journal.pgen.1010087 (PMC8979470; doi:10.1371/journal.pgen.1010087)
Supplement: S1 Text — (PDF) [file pgen.1010087.s016.pdf]

Supplemental Materials and Methods

Meiotic, genomic and evolutionary properties of crossover distribution in *Drosophila yakuba.*

Nikale Pettie, Ana Llopart and Josep M. Comeron

Generation of an updated *D. yakuba* genome sequence

The *D. yakuba* reference genome release 2.0 [1] contains ~5,400 gaps, 2,178 of them along the euchromatic genomic regions assigned to *2L*, *2R*, *3L* and *3R* chromosome arms, and the *X* and the dot chromosomes. This reference genome is based on the *D. yakuba* Tai18E2 line, which was established after multiple rounds of single pair (brother/sister) matings. In order to generate a more accurate genetic map for *D. yakuba,* and focusing on the euchromatic regions of the genome, we pursued filling gaps by PacBio (Pacific Biosciences) sequencing of the same Tai18E2 line maintained in our laboratory. High-molecular-weight DNA was extracted from adult females using standard protocols [2] and sent to the University of Washington PacBio Sequencing Services for library preparation and subsequent sequencing (https://pacbio.gs.washington.edu/). The sequences of PacBio reads used to fill gaps were corrected using illumina sequencing (see below).

PacBio contigs and gap filling

Raw PacBio reads from two PacBio SMRT cells (4 lanes) were filtered using bash5tools version 0.8.0 with a minimum read length of 500 and a minimum read score of 0.75, generating approximately 10x coverage of the *D. yakuba* reference genome. Contigs were assembled using canu v.1.8 [3], and PBJelly2 version 15.8.24 [4] was used for gap filling. All stages in the PBJelly2 pipeline were done using the defaults, with the exception of the setup and assembly stages. In the setup stage, two different values were used for the minimum size of gap to take into account that gaps in the reference sequence can either represent unknown sequences between supercontigs or otherwise between contigs (which can

be much smaller). In this regard, we also treated these two categories of gaps differently for the maximum number of nucleotides allowed to be added or subtracted, set to 5,000 or 1,000 for large and small gaps, respectively. Additionally, two different percent identity parameters were used for both gap categories, 70 (the program default) and 65 %, resulting in four outputs from PBJelly2. A python program was written to merge outputs, sequentially checking for gaps filled or improved with 70 percent identity and, if not, considering whether the gap was filled or improved with the 65 percent identity parameter. If a gap was not addressed at either percent identity, the gap sequence in the *D. yakuba* Release 2.0 was mantained.

Sample and Illumina library preparation

DNA was extracted from adult flies of Tai18E2 using a Tissue Lyser LT and the DNeasy Blood & Tissue Kit (Qiagen). After assessing concentration and quality on a NanoDrop One spectrophotometer (Thermo Scientific), the DNA was sheared using the Bioruptor UCD-200 (Diagenode) on the 'High' setting with 15 second pulse followed by 45 seconds of rest. This was repeated for a total of 24 cycles for each sample, and sheared samples were recovered using MinElute columns (Qiagen). Illumina libraries were prepared using the NEBNext DNA Library Prep Master Mix Set for Illumina (New England Biolabs). Samples were run on a 2% agarose gel for size selection (400-450 bp) and, after PCR enrichment, were purified with AMPure XP beads (Beckman-Coulter Life Sciences). Sequencing was carried out in an Illumina HiSeq 4000 at the Iowa Institute of Human Genetics (IIHG; University of Iowa) (https://medicine.uiowa.edu/humangenetics/genomics-division).

Illumina alignment pipeline

After sequencing, reads were filtered using Trimmomatic-0.36 [5] with average quality 20, trailing quality 12 and minimum length 30. Filtered reads were mapped to the updated *D. yakuba* reference

created with the PacBio reads using Bowtie2 version 2.1.0 [6], and unaligned reads were mapped again to the same reference using Stampy version 1.0.31 [7]. The aligned reads of both Bowtie2 and Stampy were then sorted and indexed using Samtools version 1.3.1 [8]. A Genome Analysis Toolkit pipeline (GATK version 3.5-0-g36282e4) was used to identify SNPs, indels and regions that needed realignment [9]. These regions were first identified by the RealignerTargetCreator and realigned with the IndelRealigner with the maximum number of reads allowed for alignment of 200,000 and all other default parameters. Finally, SNPs and indels were then called with the UnifiedGenotyper [9] with a minimum base quality score of 31; for indels we also required a minimum number of reads of 3 and a minimum fraction of reads supporting the indel of 0.51. Heterozygous sites and SNPs with allele frequency less than 0.75 and with a normalized Phred Score probability of heterozygote (het PL) < 50 were not considered at this time. FastaAlternateReferenceMaker [9] was used to create an intermediate reference genome that updated only PacBio sequences. BCFtools consensus version 1.3.1 [10] created a chain file that was used by CrossMap version 0.2.6 [11] to obtain a lift table with the new genome coordinates.

Following Lack *et al*. (2015) [12], we performed an additional round of mapping using the intermediate reference genome, which contained indels and SNPs. This second round was performed as described above using again Bowtie2 [6] and Stampy [7]. The output of CrossMap was used to filter out variants that were not located in PacBio sequences, and BCFtools consensus [10] was used to create the final chain file, which converted the coordinates of the intermediate reference to the coordinates of the new updated reference for *D. yakuba*. FastaAlternateReferenceMaker from GATK was used to create the final reference. As expected, sequence improvement with deep Illumina sequencing after two rounds of mapping allowed correcting more nucleotides of PacBio sequences (twice as many) than after one round of mapping, exemplifying the advantages of adding indels before a second mapping [12]. In all, more than 7,000 bases of the added PacBio sequences were corrected after both rounds of mapping.

A final lift table utilizing pyliftover version 0.3 was constructed to allow direct comparison between the *D. yakuba* reference genome Release 2.0 and our updated (improved) *D. yakuba* genome sequence.

Improved *D. yakuba* reference genome

Out of the original 2,178 existing gaps in the *D. yakuba* reference genome release 2.0 for *2L*, *2R*, *3L* and *3R* chromosome arms, and *X* and dot chromosomes, our PacBio and Illumina sequencing completely solved 1,483 gaps (approximately 68% of all gaps) and improved 517 (24%) additional gaps, for a total of more than 90% of gaps addressed (**Supplemental Figure 4A**). Most of the added sequence was in the centromere-proximal regions of chromosomes *2* and *3*, whereas the distribution of added sequences was more uniform in chromosomes *X* and *4* (**Supplemental Figure 4B**). We used this improved *D. yakuba* reference genome for all of our analyses.

We also explored the presence of transposable elements (TEs) in the sequences added to the reference using known *Drosophila* TEs [13] (see 'TE presence across the *D. yakuba* genome' for details). As expected, we identified an enrichment in TEs along the added sequences relative to the reference genome release 2.0 [1] sequence ($P < 2.2\times10^{-16}$).

Identification of chromosomal inversions

The presence of polymorphic inversions within *D. yakuba* is known [14, 15] and we first recognized inversions relative to the reference *D. yakuba* assembly visually as sharp peaks of crossover rates, representing inversion break points. We identified inversions relative to the reference assembly on both arms of chromosome *2* in our parental lines. The inversion on *2L* was present in all of our six parental genomes and therefore is homozygous in F_1 females. A complex inversion within an inversion was identified on *2R*, consistent with the *2Rjk* inversion [15]. This complex *2R* inversion was homozygous in two of our crosses but heterozygous in F_1 females for the cross *Sn20* x *Sn17*. Consistent with previous

studies that show that heterozygous inversions suppress crossing over [16, 17], we did not detect crossovers within this complex inversion and data for chromosome arm *2R* from the cross *Sn20* x *Sn17* was not used in any of the analyses. For homozygous inversions we identified crossover events after inverting the location of diagnostic SNPs within the inversion, but show crossover locations and rates (**Figure 2** and **Figure 3**) based on the *D. yakuba* reference assembly order for simplicity.

TE presence across the *D. yakuba* genome

To determine the distribution of TEs across the genome of *D. yakuba*, we used the sequences of known TEs from the Berkeley Drosophila Genome Project (BDGP; https://www.fruitfly.org/) [13] and aligned them to our improved *D. yakuba* reference using dc-megablast with default settings [18, 19]. The output from dc-megablast was then filtered to consider instances of different TEs aligned to the same genomic location due to sequence similarity, and the TE with the lowest Expected Value (*E*) was used. The BDGP TE database contains TEs mostly from *D. melanogaster* but also from other *Drosophila* species, including instances with the same TE characterized in different species. In these cases, we merged information to obtain a single identification per TE. Given previous studies indicating a significant presence of INE-1-like TEs in *D. yakuba* [20-24], we obtained a *D. yakuba* specific INE-1 sequence following Yang *et al*. (2006) [24]. Using the INE-1 sequences from the TE BDGP database, we identified the target sequences in the *D. yakuba* genome, aligned these target sequences to create a contig and obtained a consensus INE-1 sequence for *D. yakuba*.

To allow for a direct comparison between *D. yakuba* and *D. melanogaster,* we obtained data for TE presence in *D. melanogaster r5.3* and r*6* genome releases applying the same methodology as in *D. yakuba*. To identify differences in TE presence between *D. yakuba* and *D. melanogaster,* we applied a χ^2 test to all TEs with more than 10 copies in at least one species. Unless noted, non-overlapping 200 kb windows were used to obtain a distribution of TE presence across genomes.

Lack of evidence of P-elements in *D. yakuba*: Within the *D. melanogaster* subgroup, P-elements have been identified in *D. melanogaster* and more recently in *D. simulans,* but not in the sister species *D. sechellia* or *D. mauritiana* [25-29]. Initial analyses of TE distribution across the *D. yakuba* genome recovered a few alignments to P-elements, possibly suggesting a recent invasion of P-elements in *D. yakuba*. We expanded this study by using Illumina reads from multiple lines of *D. yakuba* and Bowtie2 to recover alignments to the *D. melanogaster* P-element. The alignments to the P-element were consistently limited to only small regions of exons 1 and 3. The absence of alignments to exons 0 and 2 suggests that there is no active P-element in *D. yakuba* because all four exons are required for transposition (reviewed in [30]). Furthermore, alignments to exon 1 cluster in an approximately 202 bp portion in the center of the exon and all alignments to exon 3 cluster in a narrow region of ~114 bp. Notably, this same small region shows high sequence similarity with INE-1, and alignments of Illumina reads from an experimental profiling of *Drosophila* microRNAs [31-33] also show mapping to the regions of similarlity between INE-1 and P-elements. Combined, our analyses do not support the presence of P-elements in *D. yakuba* in the recent past. Instead, the results indicate that recovered partial alignments are the consequence of sequence similarity with other TEs, including INE-1.

Satellite repeats and TE presence in heterochromatic regions

The *D. yakuba* reference genome does not include heterochromatin and the sequences of centromere and telomere regions are unknown. Therefore, we used PacBio reads and assumed that reads that do not align to the updated *D. yakuba* reference genome (nuclear and mitochondrial) are mostly heterochromatic. To determine if satellite repeats enriched near centromeres in *D. melanogaster* were also enriched in *D. yakuba*, we investigated the presence of k-mers in PacBio reads of *D. yakuba* and *D. melanogaster*. Raw PacBio reads for *D. melanogaster* were obtained from Kim *et al.* (2014) [34] and

analyzed and filtered using the same approach as that in *D. yakuba*. PacBio reads were aligned with BLASR [35] to our *D. yakuba* reference and to *D. melanogaster* r5.3 and r6 references. After alignment, mapped and unmapped reads were considered euchromatic and heterochromatic, respectively, and cut into 125 bp sequences to find kmers with k-seek [36, 37]. For each repeat, a χ^2 test was performed to compare the ratio of kmers aligned to heterochromatic PacBio reads in *D. yakuba* and *D. melanogaster* and the Benjamini-Hochberg method was applied to correct for multiple tests [38].

To examine the degree of TE enrichment in heterochromatic or euchromatic regions of *D. yakuba* and *D. melanogaster*, PacBio reads were used to create BLAST databases. The BDGP TE database was then aligned to these databases using dc-megablast. A goodness of fit test was performed to compare the number of alignments in the heterochromatic and euchromatic reads of each species, and the Benjamini-Hochberg correction was applied.

Short DNA motifs and crossover localization

We investigated the presence of short DNA motifs previously reported to be predictive of changes in crossover rates across the *D. melanogaster* genome [39-41]. For each of these motifs, we used FIMO [42] and the position probability matrix (PPM) reported in [39] to annotate motif distribution across the *D. yakuba* genome. To investigate whether motifs were enriched near crossover events, we took into account the variable distance between diagnostic SNPs flanking crossover events and the unknown precise location of crossovers. We, therefore, created three datasets of sequences containing crossovers defined by the different distance between diagnostic flanking SNPs (1 kb or less, 3 kb or less, and 5 kb or less) and, unless noted, used the dataset of 1 kb (or less) by default. For this analysis, chromosome arm *2R* was not included due to the smaller sample size relative to the other chromosome arms. To quantify enrichment, we applied FIMO to a set of randomly chosen genomic sequences of identical length to

each of the sequences with crossovers, and this random selection was repeated 1,000 times to create a null expectation.

References

1. Drosophila 12 Genomes Consortium. Evolution of genes and genomes on the Drosophila phylogeny. Nature. 2007;450:203. doi: 10.1038/nature06341.
2. Ashburner M. Drosophila: A Laboratory Manual. New York: Cold Spring Harbor Laboratory Press; 1989.
3. Koren S, Walenz BP, Berlin K, Miller JR, Bergman NH, Phillippy AM. Canu: scalable and accurate long-read assembly via adaptive k-mer weighting and repeat separation. Genome Res. 2017;27(5):722-36. Epub 2017/03/17. doi: 10.1101/gr.215087.116. PubMed PMID: 28298431; PubMed Central PMCID: PMCPMC5411767.
4. English AC, Richards S, Han Y, Wang M, Vee V, Qu J, et al. Mind the Gap: Upgrading Genomes with Pacific Biosciences RS Long-Read Sequencing Technology. PLOS ONE. 2012;7(11):e47768. doi: 10.1371/journal.pone.0047768.
5. Bolger AM, Lohse M, Usadel B. Trimmomatic: a flexible trimmer for Illumina sequence data. Bioinformatics. 2014;30(15):2114-20. doi: 10.1093/bioinformatics/btu170. PubMed PMID: PMC4103590.
6. Langmead B, Salzberg SL. Fast gapped-read alignment with Bowtie 2. Nature Methods. 2012;9:357. doi: 10.1038/nmeth.1923.
7. Lunter G, Goodson M. Stampy: A statistical algorithm for sensitive and fast mapping of Illumina sequence reads. Genome Res. 2011;21(6):936-9. doi: 10.1101/gr.111120.110. PubMed PMID: PMC3106326.
8. Li H. A statistical framework for SNP calling, mutation discovery, association mapping and population genetical parameter estimation from sequencing data. Bioinformatics. 2011;27(21):2987-93. doi: 10.1093/bioinformatics/btr509. PubMed PMID: PMC3198575.
9. DePristo MA, Banks E, Poplin R, Garimella KV, Maguire JR, Hartl C, et al. A framework for variation discovery and genotyping using next-generation DNA sequencing data. Nat Genet. 2011;43(5):491-8. Epub 2011/04/12. doi: 10.1038/ng.806. PubMed PMID: 21478889; PubMed Central PMCID: PMCPMC3083463.
10. Li H, Handsaker B, Wysoker A, Fennell T, Ruan J, Homer N, et al. The Sequence Alignment/Map format and SAMtools. Bioinformatics. 2009;25(16):2078-9. doi: 10.1093/bioinformatics/btp352. PubMed PMID: PMC2723002.

11. Zhao H, Sun Z, Wang J, Huang H, Kocher JP, Wang L. CrossMap: a versatile tool for coordinate conversion between genome assemblies. Bioinformatics. 2014;30(7):1006-7. Epub 2013/12/20. doi: 10.1093/bioinformatics/btt730. PubMed PMID: 24351709; PubMed Central PMCID: PMCPMC3967108.
12. Lack JB, Cardeno CM, Crepeau MW, Taylor W, Corbett-Detig RB, Stevens KA, et al. The *Drosophila* Genome Nexus: A Population Genomic Resource of 623 *Drosophila melanogaster* Genomes, Including 197 From a Single Ancestral Range Population. Genetics. 2015;199(4):1229-41. Epub 2015/01/30. doi: 10.1534/genetics.115.174664. PubMed PMID: 25631317; PubMed Central PMCID: PMCPMC4391556.
13. Kaminker JS, Bergman CM, Kronmiller B, Carlson J, Svirskas R, Patel S, et al. The transposable elements of the Drosophila melanogaster euchromatin: a genomics perspective. Genome Biology. 2002;3(12):research0084.1. doi: 10.1186/gb-2002-3-12-research0084.
14. Ranz JM, Maurin D, Chan YS, von Grotthuss M, Hillier LW, Roote J, et al. Principles of Genome Evolution in the Drosophila melanogaster Species Group. PLoS biology. 2007;5(6):e152. doi: 10.1371/journal.pbio.0050152.
15. Lemeunier F, Ashburner M, Thoday JM. Relationships within the melanogaster species subgroup of the genus Drosophila (Sophophora) - II. Phylogenetic relationships between six species based upon polytene chromosome banding sequences. Proceedings of the Royal Society of London Series B Biological Sciences. 1976;193(1112):275-94. doi: 10.1098/rspb.1976.0046.
16. Sturtevant AH, Beadle GW. The Relations of Inversions in the X Chromosome of Drosophila Melanogaster to Crossing over and Disjunction. Genetics. 1936;21(5):554-604. PubMed PMID: 17246812.
17. Sturtevant AH. A Case of Rearrangement of Genes in Drosophila. Proc Natl Acad Sci U S A. 1921;7(8):235-7. doi: 10.1073/pnas.7.8.235. PubMed PMID: 16576597.
18. Altschul SF, Gish W, Miller W, Myers EW, Lipman DJ. Basic local alignment search tool. Journal of Molecular Biology. 1990;215(3):403-10. doi: https://doi.org/10.1016/S0022-2836(05)80360-2.
19. Morgulis A, Coulouris G, Raytselis Y, Madden TL, Agarwala R, Schäffer AA. Database indexing for production MegaBLAST searches. Bioinformatics. 2008;24(16):1757-64. doi: 10.1093/bioinformatics/btn322.
20. Kapitonov VV, Jurka J. Molecular paleontology of transposable elements in the *Drosophila melanogaster* genome. Proceedings of the National Academy of Sciences. 2003;100(11):6569-74. doi: 10.1073/pnas.0732024100.

21. Petrov DA, Fiston-Lavier A-S, Lipatov M, Lenkov K, González J. Population genomics of transposable elements in Drosophila melanogaster. Molecular biology and evolution. 2011;28(5):1633-44. Epub 12/16. doi: 10.1093/molbev/msq337. PubMed PMID: 21172826.
22. Yang H-P, Barbash DA. Abundant and species-specific DINE-1 transposable elements in 12 Drosophila genomes. Genome Biology. 2008;9(2):R39. doi: 10.1186/gb-2008-9-2-r39.
23. Lu J, Clark AG. Population dynamics of PIWI-interacting RNAs (piRNAs) and their targets in Drosophila. Genome Research. 2010;20(2):212-27. doi: 10.1101/gr.095406.109.
24. Yang H-P, Hung T-L, You T-L, Yang T-H. Genomewide Comparative Analysis of the Highly Abundant Transposable Element *DINE-1* Suggests a Recent Transpositional Burst in *Drosophila yakuba*. Genetics. 2006;173(1):189-96. doi: 10.1534/genetics.105.051714.
25. Kofler R, Hill T, Nolte V, Betancourt AJ, Schlotterer C. The recent invasion of natural Drosophila simulans populations by the P-element. Proc Natl Acad Sci U S A. 2015;112(21):6659-63. Epub 2015/05/13. doi: 10.1073/pnas.1500758112. PubMed PMID: 25964349; PubMed Central PMCID: PMCPMC4450375.
26. Kidwell MG. Evolution of hybrid dysgenesis determinants in Drosophila melanogaster. Proc Natl Acad Sci U S A. 1983;80(6):1655-9. Epub 1983/03/01. doi: 10.1073/pnas.80.6.1655. PubMed PMID: 6300863; PubMed Central PMCID: PMCPMC393661.
27. Anxolabehere D, Kidwell MG, Periquet G. Molecular characteristics of diverse populations are consistent with the hypothesis of a recent invasion of Drosophila melanogaster by mobile P elements. Mol Biol Evol. 1988;5(3):252-69. Epub 1988/05/01. doi: 10.1093/oxfordjournals.molbev.a040491. PubMed PMID: 2838720.
28. Engels WR. The origin of P elements in Drosophila melanogaster. Bioessays. 1992;14(10):681-6. Epub 1992/10/01. doi: 10.1002/bies.950141007. PubMed PMID: 1285420.
29. Daniels SB, Peterson KR, Strausbaugh LD, Kidwell MG, Chovnick A. Evidence for horizontal transmission of the P transposable element between Drosophila species. Genetics. 1990;124(2):339-55. Epub 1990/02/01. PubMed PMID: 2155157; PubMed Central PMCID: PMCPMC1203926.
30. Kelleher ES. Reexamining the *P*-Element Invasion of *Drosophila melanogaster* Through the Lens of piRNA Silencing. Genetics. 2016;203(4):1513-31. doi: 10.1534/genetics.115.184119.
31. Mohammed J, Flynt AS, Panzarino AM, Mondal MMH, DeCruz M, Siepel A, et al. Deep experimental profiling of microRNA diversity, deployment, and evolution across the Drosophila genus. Genome Res. 2018;28(1):52-65. doi: 10.1101/gr.226068.117.

32. Mohammed J, Bortolamiol-Becet D, Flynt AS, Gronau I, Siepel A, Lai EC. Adaptive evolution of testis-specific, recently evolved, clustered miRNAs in Drosophila. RNA. 2014;20(8):1195-209. doi: 10.1261/rna.044644.114.
33. Shpiz S, Ryazansky S, Olovnikov I, Abramov Y, Kalmykova A. Euchromatic Transposon Insertions Trigger Production of Novel Pi- and Endo-siRNAs at the Target Sites in the Drosophila Germline. PLOS Genetics. 2014;10(2):e1004138. doi: 10.1371/journal.pgen.1004138.
34. Kim KE, Peluso P, Babayan P, Yeadon PJ, Yu C, Fisher WW, et al. Long-read, whole-genome shotgun sequence data for five model organisms. Sci Data. 2014;1:140045-. doi: 10.1038/sdata.2014.45. PubMed PMID: 25977796.
35. Chaisson MJ, Tesler G. Mapping single molecule sequencing reads using basic local alignment with successive refinement (BLASR): application and theory. BMC Bioinformatics. 2012;13(1):238. doi: 10.1186/1471-2105-13-238.
36. Wei KH-C, Grenier JK, Barbash DA, Clark AG. Correlated variation and population differentiation in satellite DNA abundance among lines of *Drosophila melanogaster*. Proceedings of the National Academy of Sciences. 2014;111(52):18793-8. doi: 10.1073/pnas.1421951112.
37. Wei KH, Lower SE, Caldas IV, Sless TJS, Barbash DA, Clark AG. Variable Rates of Simple Satellite Gains across the Drosophila Phylogeny. Mol Biol Evol. 2018;35(4):925-41. Epub 2018/01/24. doi: 10.1093/molbev/msy005. PubMed PMID: 29361128; PubMed Central PMCID: PMCPMC5888958.
38. Benjamini Y, Hochberg Y. Controlling the false discovery rate: a practical and powerful approach to multiple testing. Journal of the Royal Statistical Society, Series B 1995;57(1):289-300. doi: 10.1016/s0166-4328(01)00297-2 PubMed Central PMCID: PMC11682119.
39. Adrian AB, Corchado JC, Comeron JM. Predictive Models of Recombination Rate Variation across the *Drosophila melanogaster* Genome. Genome Biology and Evolution. 2016;8(8):2597-612. doi: 10.1093/gbe/evw181.
40. Howie JM, Mazzucco R, Taus T, Nolte V, Schlötterer C. DNA Motifs Are Not General Predictors of Recombination in Two Drosophila Sister Species. Genome Biology and Evolution. 2019;11(4):1345-57. doi: 10.1093/gbe/evz082.
41. Comeron JM, Ratnappan R, Bailin S. The Many Landscapes of Recombination in *Drosophila melanogaster*. PLOS Genetics. 2012;8(10):e1002905. doi: 10.1371/journal.pgen.1002905.
42. Bailey TL, Boden M, Buske FA, Frith M, Grant CE, Clementi L, et al. MEME Suite: tools for motif discovery and searching. Nucleic Acids Research. 2009;37(suppl_2):W202-W8. doi: 10.1093/nar/gkp335.
